# Loss of FADD and Caspases Affects the Response of T-Cell Leukemia Jurkat Cells to Anti-Cancer Drugs

**DOI:** 10.3390/ijms22052702

**Published:** 2021-03-07

**Authors:** Zuzana Mrkvová, Michaela Portešová, Iva Slaninová

**Affiliations:** Department of Biology, Faculty of Medicine, Masaryk University, Kamenice 5, Building A6, 625 00 Brno, Czech Republic; Zuzana.Mrkvova@seznam.cz (Z.M.); michaela.portesova@seznam.cz (M.P.)

**Keywords:** apoptosis, cancer, caspase, cell death, FADD, leukemia, necroptosis, RIP1, RIP3, ripoptosome

## Abstract

Programmed cell death (PCD) pathways play a crucial role in the response of cancer cells to treatment. Their dysregulation is one of the cancer hallmarks and one of the reasons of drug resistance. Here, we studied the significance of the individual members of PCD signaling pathways in response to treatment with common anti-cancer drugs using the T-cell leukemia Jurkat cells with single or double knockouts of necroptosis and/or apoptosis genes. We identified apoptosis as the primary cell death pathway upon anti-cancer drugs treatment. The cells with knocked out either Fas-associated protein with death domain (FADD) or all executioner caspases were resistant. This resistance could be partially overcome by induction of RIP1-dependent necroptosis through TNFR1 activation using combined treatment with TNF-α and smac mimetic (LCL161). RIP1 was essential for cellular response to TNF-α and smac mimetic, but dispensable for the response to anti-cancer drugs. Here, we demonstrated the significance of FADD and executioner caspases in carrying out programmed cell death upon anti-cancer drug treatments and the ability of combined treatment with TNF-α and smac mimetic to partially overcome drug resistance of FADD and/or CASP3/7/6-deficient cells via RIP1-dependent necroptosis. Thus, a combination of TNF-α and smac mimetic could be a suitable strategy for overcoming resistance to therapy in cells unable to trigger apoptosis.

## 1. Introduction

Programmed cell death (PCD) plays a crucial role in the multicellular organism development and in the cellular response to stress conditions including treatment with cytostatics. Failure of the cell death mechanisms exacerbates outcomes of cancer therapy. To date, several signal-transduction pathways involved in PCD are recognized [1]. Apoptosis, PCD-dependent on caspases, is the best known one. Initiator caspases, caspase 8, and caspase 10, propagate a death signal in response to a death receptor activation (extrinsic pathway), and caspase 9 in response to mitochondrial outer membrane permeabilization (MOMP, intrinsic pathway) [2]. Caspase 3 is the main executioner caspase that, upon activation by initiator caspases, cleaves a wide panel of intracellular proteins mediating cellular breakdown [1]. However, caspase 6 and/or caspase 7 also act as executioner caspases and can replace caspase 3 [3]. Recently, information about necroptosis and its role in the physiology and pathology of the cell and the whole organism has appeared [4,5]. Necroptosis is mediated by necrosome, a complex of receptor-interacting serine-threonine kinase 1 (RIP1) and 3 (RIP3), which activates mixed lineage kinase domain-like protein (MLKL) and shows morphological features of necrosis [6]. The best characterized signal transduction cascade resulting in necroptotic cell death is initiated by TNFR1 (tumor necrosis factor receptor 1) ligation in the presence of caspase inhibitors and/or smac mimetics (SM, inhibitors of cIAPs).

**Figure 1:**
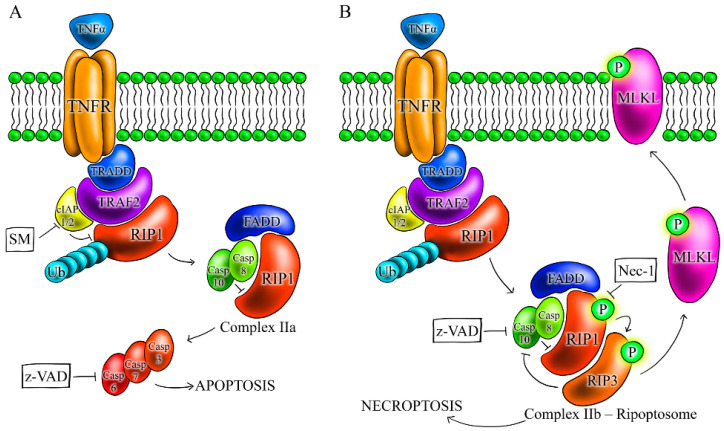
Scheme of complexes formed in response to TNFα stimulation of tumor necrosis factor receptor 1(TNFR1). (**A**) Activation of TNFR1 by TNF-α leads to the trimerization of TNFR1 and to the formation of complex I containing TRADD, TRAF2, receptor-interacting serine-threonine kinase 1 (RIP1), cIAP1/2, and E3 ligases that mediate the ubiquitination of RIP1. Internalization of complex I, deubiquitination of RIP1, and recruitment of FAS-associated death domain protein (FADD) and caspase 8 form complex IIa, which may activate apoptosis through caspase 8. Active caspase 8 cleaves and inactivates RIP1 and necroptosis. (**B**) When caspase 8 is inactivated, complex IIb (ripoptosome), which includes RIP1, RIP3, mixed lineage kinase domain-like protein (MLKL), caspase 8, and FADD, is formed, and RIP1, RIP3, and MLKL are activated by phosphorylation and necroptosis is induced. **→**—activation, **┴**—inhibition.

RIP1 plays a key role in cell decision about death and life [7]. The induction of apoptosis or necroptosis or triggering prosurvival NF-κB pathway upon stimulation of TNFR1 by TNF-α depends on the ubiquitination of RIP1 kinase and on the activity of caspase 8. RIP1 seems to be an important member in regulation of all abovementioned pathways since it is present in each of the three key complexes that mediate downstream signaling of TNFR1. Complex I, which is associated with the intracellular domain of TNFR1 consists of TRADD, RIP1, TRAF2, and cIAP1/2 [8]. In the absence of survival signaling, this membrane-associated complex I transits to cytosolic complex IIa containing FADD, RIP1, and caspase 8, which is activated and triggers apoptosis (Figure 1) [8,9]. If apoptosis is blocked and RIP1 is deubiquitinated and interacts with RIP3, alternative complex IIb (necrosome) responsible for necroptosis is formed [10,11,12]. Caspase 8 and FADD, found in both complexes (IIa and IIb), are critical mediators of apoptosis [7]. Loss of cIAP1 and cIAP2 due to SM (smac mimetic) or etoposide treatment, induces association of RIP1, FADD, and caspase 8 and forms cytoplasmic complex ripoptosome even in the absence of death receptor stimulation. Necroptosis or RIP1 kinase-dependent apoptosis could be induced by this complex (Figure 1) [13,14].

Deregulation of PCD signaling plays an important role not only in cancer progression but also in the response to treatment. Evasion of apoptosis is one of the cancer hallmarks, and its failure could lead to drug resistance [15]. Cancer cells die by programmed cell death upon treatment with anti-cancer drugs. Therefore, better understanding of the cell death signaling pathways and the role of their key players upon treatment with anti-cancer drugs prove to be helpful in finding more efficient cancer therapy.

Since several signaling pathways are involved in PCD, the aim of the present study was to determine which of these pathways is triggered by conventional anti-cancer drugs and which molecules of cell death signaling pathways are involved in these processes. For this purpose, we used the T-leukemia Jurkat cells with the single or double knockouts for necroptosis and/or apoptosis genes, created by the CRISPR-Cas9 editing system, and inhibitors of apoptosis and/or necroptosis.

The results show that all tested anti-cancer drugs induce caspase-dependent cell death. Knockout of all three executioner caspases renders the cells immortal. However, the new and much more interesting finding is that the FADD protein is indispensable for Jurkat cell death. In addition, we demonstrated that the cells resistant to anti-cancer drugs (CASP3/7/6 and FADD-deficient cells) are sensitive to treatment with TNF-α/SM, which triggers RIP1-dependent necroptosis. These results revealed the important role of FADD and executioner caspases in cell death execution upon anti-cancer drugs treatment and suggest the possibility to partially overcome resistance of CASP3/7/6 and FADD-deficient cells by combined treatment with TNF-α and SM. 

## 2. Results

### 2.1. FADD and Executioner Caspases Are Indispensable for the Cell Death upon Anti-Cancer Drugs Treatment

To reveal what PCD modality is triggered in Jurkat cells upon treatment with anti-cancer drugs, we studied the effect of a spectrum of anti-cancer drugs with different mechanisms of action. The cell viability was investigated in WT cells and in the cells with knocked out one or more genes, whose products are involved in programmed cell death pathways (RIP1, RIP3, CASP8, CASP3, CASP6, CASP3/7, CASP3/7/6, MLKL, MLKL/CASP8, FADD, MLKL/FADD, and RIP3/FADD) using PI-exclusion assay.

**Figure 2:**
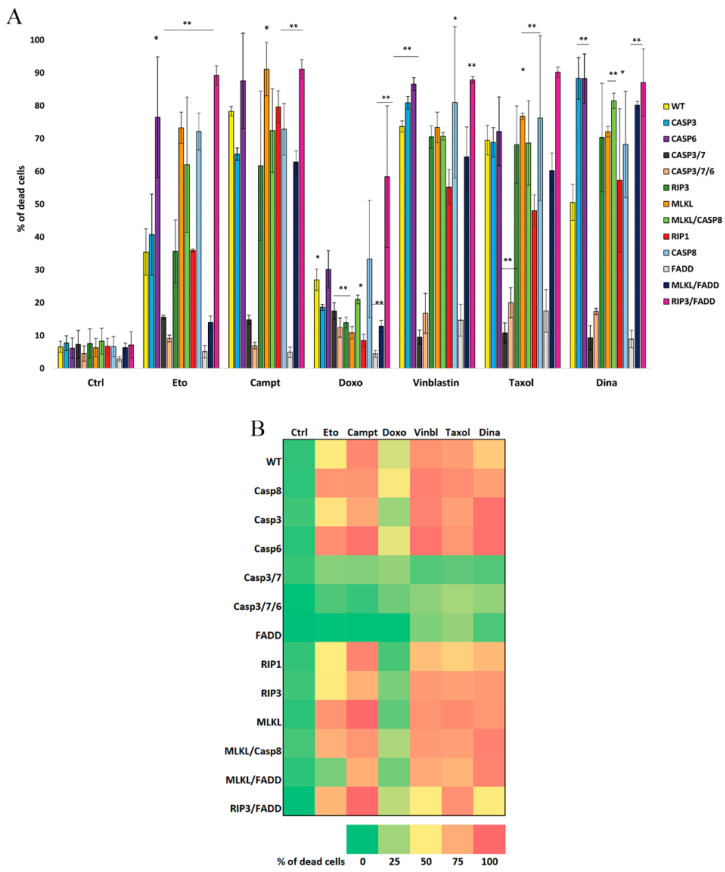
Cytotoxicity of anti-cancer drugs on knocked out cells. (**A**) Flow cytometry analysis of propidium iodide (PI)-stained Jurkat cells with knocked out one or more genes, whose products are involved in programmed cell death pathways showing percentage of dead cells after 48 h treatment with anti-cancer drugs camptothecin (Campt, 1 μM), etoposide (Eto, 5 μM), doxorubicin (Doxo, 0.15 μM), vinblastine (vinbl, 0.1 μM), Taxol (0.1 μM), and dinaciclib (Dina, 40 nM). When compared to wild type (WT) cells, statistically significant difference in drugs toxicity to knocked out cells was observed. Data are means ± S.E.M. of three independent experiments. * *p* < 0.01, ** *p* < 0.005 WT vs. knocked out cells. (**B**) Graph summarizing results of PI exclusion assays. The cells with disrupted genes for single caspases died more or equally to WT. Only caspase 3-deficient (CASP3) cells died less upon Campt and Doxo treatment. Cells with individually knocked out RIP1 or RIP3 died less than cells with knocked out MLKL or combination of apoptotic and non-apoptotic genes (RIP3/FADD, MLKL/FADD, and MLKL/CASP8).

The first category of tested drugs was topoisomerase I (camptothecin, Campt) and topoisomerase II (doxorubicin (Doxo) and etoposide (Eto)) inhibitors. Upon Eto (5 μM) treatment, approximately 36% of the WT cells died. CASP3/7/6-, CASP3/7-, MLKL/FADD-, and FADD-deficient cells were the least susceptible to cell death. Interestingly, we observed higher percentages of dead cells in the case of CASP6-, MLKL-, CASP8-, and RIP3/FADD-deficient cells than in the WT cells. CASP3-, RIP3-, MLKL/CASP8-, and RIP1-deficient cells did not show a statistically significant difference in mortality compared to WT cells. Campt (1 μM) treatment killed approximately 78% of the WT cells. CASP3/7/6-, CASP3/7-, and FADD-deficient cells died the least. Compared to WT cells CASP3-, and CASP8-deficient cells revealed significantly lower cell mortality. MLKL- and RIP3/FADD- deficient cells were more prone to die than WT cells. CASP6-, RIP3-, MLKL/CASP8-, RIP1-, and CASP8-deficient cells did not show a significant difference in death rate compared to WT cells. Doxo (0.15 μM) was slightly toxic even to WT cells (27%), nevertheless, the percentage of dead FADD-, RIP1-, CASP3-, CASP 3/7-, CASP3/7/6-, RIP3-, MLKL-, MLKL/CASP8-, and MLKL/FADD-deficient cells was lower than of WT cells. CASP6- and CASP8-deficient cells died approximately as much as WT cells. The most sensitive were RIP3/FADD-deficient cells (Figure 2A).

The second category of tested cytostatics were microtubule inhibitors vinblastine (vinbl) and Taxol. Upon vinbl (0.1 μM) treatment, approximately 74% of the WT cells died. CASP3/7/6-, CASP3/7-, and FADD-deficient cells were the least prone to die. MLKL/CASP8- and RIP1-deficient cells revealed lower mortality than WT cells. CASP3-, CASP6-, CASP8-, and RIP3/FADD-deficient cells died more than the WT cells. RIP3-, MLKL-, and MLKL/FADD-deficient cells did not show a statistically significant difference compared to WT cells. Taxol (0.1 μM) killed approximately 70% of the WT cells. CASP3/7/6-, CASP3/7-, and FADD-deficient cells died the least. RIP1- and MLKL/FADD-deficient cells showed lower mortality than WT cells and CASP3-, CASP6-, RIP3-, MLKL-, MLKL/CASP8-, and CASP8-deficient cells showed similar mortality to WT cells. Only RIP3/FADD-deficient cells were prone to die significantly more than WT cells (Figure 2A).

The last tested compound was CDK inhibitor dinaciclib (Dina 40 nM). Approximately 50% of the WT cells died upon Dina treatment. All other cell lines, except for FADD-, CASP3/7-, and CASP3/7/6-deficient cells, died more than WT (Figure 2A). 

Collectively, these results show that the most resistant cell lines to anti-cancer drug treatments are FADD-, CASP3/7/6-, and CASP3/7-deficient cells. A graph summarizing all data shows that cells with disrupted genes for single caspases were equally or even more susceptible to die than WT. Only CASP3-deficient cells showed lower percentage of dead cells upon Campt and Doxo treatment. Cells with individually knocked out RIP1 and/or RIP3 gene were less prone to dying than cells with knocked out MLKL or combination of apoptotic and non-apoptotic genes. Interestingly, RIP3/FADD-deficient cell line was the most sensitive (Figure 2B). 

### 2.2. TNF-α and Smac Mimetic Overcome Drug Resistance of CASP 3/7/6-, CASP3/7-, and FADD-Deficient Cells

**Figure 3:**
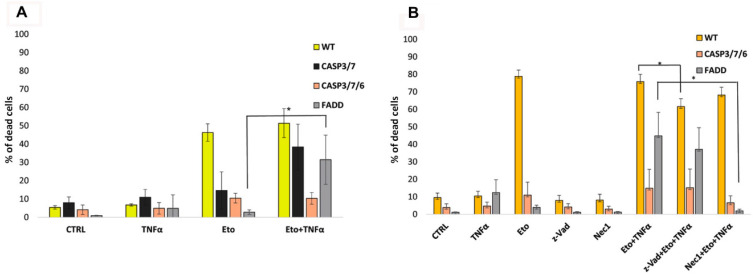
Effect of TNF-α on Eto cytotoxicity. (**A**) Flow cytometry analysis of PI-stained cells showing percentage of the dead cells after 48 h treatment with Eto (5 μM) and TNF-α (10 ng/mL). TNF-α potentiated cytotoxicity of Eto in FADD- and CASP3/7-deficient cells. It had nearly no effect on CASP3/7/6-deficient cells. * *p* < 0.01. Eto vs. Eto/TNF-α. (**B**) Pretreatment with the pancaspase inhibitor (z-VAD, 20 μM) and/or RIP1 inhibitor necrostatin-1 (Nec-1, 20 μM) decreased dying of WT cells, while dying of CASP3/7/6- and FADD-deficient cells were affected only by Nec-1. Data are means ± S.E.M. of three independent experiments. Comparisons of Eto + TNF-α vs. inhibitors + Eto +TNF-α are shown.

Since CASP3/7-, CASP 3/7/6-, and FADD-deficient cells were the most resistant to all of the abovementioned treatments, we tried to induce their cell death by activating TNFR1 death receptor pathway using cytokine TNF-α. Therefore, we studied the effect of TNF-α on cytotoxicity of Eto. WT cells died extensively upon Eto treatment and a combination of Eto with TNF-α (10 ng/mL) did not significantly increase cell death. TNF-α alone slightly affected viability of FADD-deficient cells only. It significantly potentiated cytotoxicity of Eto in FADD- and CASP3/7-deficient cells, while it had nearly no synergistic effect on CASP3/7/6-deficient cells (Figure 3A). This indicates the importance of caspase 6 for TNFR1-mediated cell death signaling in the absence of other executioner caspases. 

To better recognize the mechanism of the cell death, we preincubated cells with the pancaspase inhibitor (z-VAD; 20 μM) and/or RIP1 inhibitor necrostatin-1 (Nec-1; 20 μM) and subsequently treated them with TNF-α and Eto. z-VAD reduced dying of WT cells more significantly than Nec-1, while dying of CASP3/7/6- and FADD-deficient cells was significantly decreased only by Nec-1, but FADD-deficient cells partially also by z-VAD. These results indicate that the primary type of cell death is probably apoptosis, which is triggered in WT cells. This assumption is supported by the fact that cells lacking executioner caspases (CASP3/7/6-deficient cells) extensively resisted cell death, while FADD deficiency enabled triggering of RIP1-dependent cell death (Figure 3B). This indicates that the cells deficient in executioner caspases cannot switch from apoptosis to necroptosis upon this treatment.

**Figure 4:**
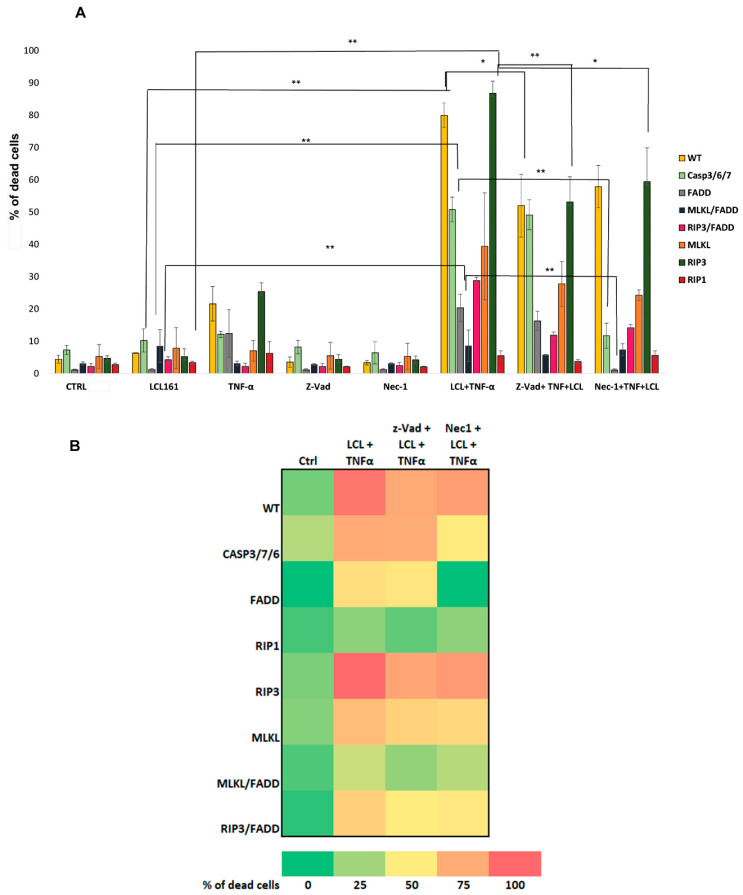
Effect of smac mimetic (LCL161) and TNF-α. (**A**) Flow cytometry analysis of PI-stained cells showing percentage of the dead cells after 48 h treatment with LCL161 (10 μM) and TNF-α (10 ng/mL) and the effect of the pretreatment with the pancaspase inhibitor (z-VAD, 20 μM) and/or RIP1 inhibitor necrostatin-1 (Nec-1, 20 μM). LCL161 alone was nontoxic and TNF-α revealed a slight toxicity to WT cells and CASP3/7/6-, FADD-, and RIP3-deficient cells. The combination of these compounds significantly increased dying of the WT cells, CASP3/7/6, RIP3-deficient cells and also slightly of FADD-, RIP3/FADD-, and MLKL-deficient cells. RIP1-deficient cells almost were not affected by this treatment. z-VAD in combination with LCL161 and TNF-α did not affect CASP3/7/6- and FADD-deficient cells mortality, while Nec-1 effect was significant. Dying of WT cells and RIP3/FADD-, MLKL-, and RIP3-deficient cells was affected by both inhibitors. MLKL/FADD-deficient cells revealed weak sensitivity to treatment with LCL161/TNF-α, which was only slightly affected by z-VAD but not by Nec-1. Data are means ± S.E.M. of three independent experiments. * *p* < 0.01, ** *p* < 0.005 LCL161 vs. LCL161/TNF-α; LCL161 +TNF-α vs. inhibitors + LCL161/TNF-α. (**B**) Graph summarizing results of the effect of z-VAD and Nec-1 on combined treatment with LCL161/TNF-α.

In the following experiments, we tried another way to hit resistant cells. We took advantage of SM (LCL161) ability to potentiate induction of apoptosis and/or necroptosis by inhibition of cIAPs. We treated cells with combination of LCL161 (10 μM) and TNF-α (10 ng/mL) and compared the response of the cells that were the least prone to die, i.e., CASP3/7/6- and FADD-deficient cells, and the cells with knocked out genes for necroptosis, i.e., MLKL-, RIP3-, RIP1-, MLKL/FADD-, and RIP3/FADD-deficient cells, with WT cells. Despite LCL161 alone was not toxic and TNF-α revealed only slight toxicity to WT cells and CASP3/7/6-, FADD-, and RIP3-deficient cells, the combination of these compounds significantly increased the percentage of dead cells in the case of WT cells and CASP3/7/6- and RIP3-deficient cells and slightly in the case of FADD-, RIP3/FADD-, and MLKL-deficient cells. LCL161 was slightly toxic to MLKL/FADD-deficient cells and TNF-α did not potentiate its toxicity, while potentiation was significant in RIP3/FADD-deficient cells. Interestingly, RIP1-deficient cells were almost not affected by this treatment, which indicates the importance of RIP1 kinase in the response to LCL161 and TNF-α treatment (Figure 4).

To find out what cell death modality was induced upon the combined treatment with TNF-α and LCL161, we again preincubated cells with the inhibitors z-VAD and/or Nec-1. z-VAD in combination with LCL161 and TNF-α did not affect CASP3/7/6-deficient cells mortality, while Nec-1 effect was significant (proportion of dead cells decreased from 51% to 12%). We observed similar responses in FADD-deficient cells (cell death decreases from 20% to 1%). These results suggest that TNF-α and LCL161 co-treatment induces RIP1-dependent cell death in CASP3/7/6- and FADD-deficient cells. This finding is in accordance with our previous results showing resistance of RIP1-deficient cells to LCL161/TNF-α treatment. Dying of WT cells and RIP3/FADD- and MLKL-deficient cells was affected by both inhibitors, which indicates either mixed death phenotype or RIP1-dependent apoptosis. RIP3-deficient cells were the most prone to die and their dying was more affected by z-VAD, which indicates induction of apoptosis. Weak sensitivity of MLKL/FADD-deficient cells to treatment with LCL161/TNF-α was only slightly affected by z-VAD but not by Nec-1, showing importance of both FADD and MLKL for cell death induction (Figure 4A,B). 

Collectively, these results show that TNF-α potentiates the effect of SM (LCL161) in WT cells and cells with affected apoptotic and, to less extent, necroptotic pathway. RIP1, but not MLKL or RIP3 alone, plays a key role in cell death triggered by this treatment. Moreover, combined knockout of FADD and MLKL but not FADD and RIP3 affects the cellular mortality upon LCL161/TNF-α treatment indicating inhibitory effect of RIP3 on apoptosis in the absence of FADD. Different reactions of RIP3/FADD- and MLKL/FADD-deficient cells can also indicate induction of apoptosis in RIP3-deficient cells.

### 2.3. Apoptosis Is the Primary Cell Death in WT Cells, While FADD- and CASP3/7/6-Deficient Cells Die by RIP1-Dependent Necroptosis

**Figure 5:**
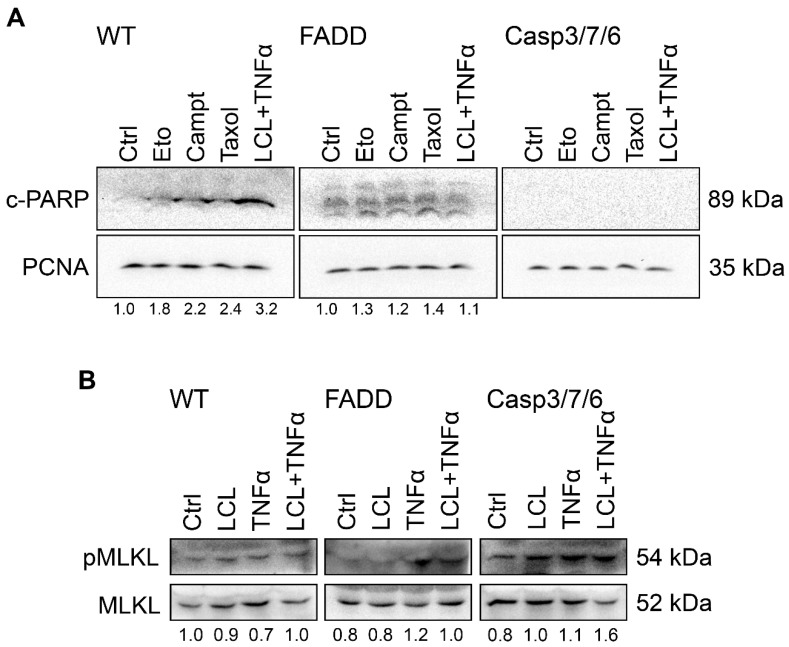
Western blot analysis of markers of apoptosis (cleaved PARP; c-PARP) and necroptosis (pMLKL). (**A**) c-PARP detection after 3 h treatment of WT cells and FADD-, and CASP3/7/6-deficient cells with Eto (5 μM), Campt (1 μM), Taxol (0.1 μM), and LCL161/TNF-α (10 μM; 10 nM). In WT cells, c-PARP was present upon treatment with all compounds, while in FADD-deficient cells, basal level of c-PARP was present even in control cells. In CASP3/7/6-deficient cells, no c-PARP signal was detected. PCNA was used as a loading control. Total cell lysates were separated on 10% gels. Numeric values represent the ratio of band densities of c-PARP normalized to the corresponding PCNA and the control normalized to the corresponding PCNA. (**B**) Detection of MLKL and pMLKL necroptosis marker in WT cells and CASP3/7/6- and FADD-deficient cells after 24 h treatment with LCL161, TNF-α, and LCL161/TNF-α using Western blot analysis. Total cell lysates were separated on 10% gels. Numeric values represent the ratio of band densities of pMLKL to MLKL.

The abovementioned results indicate that T-cell leukemia Jurkat cells die by apoptosis upon treatment with conventional anti-cancer drugs. FADD- and executioner caspases-deficient cells are resistant to this treatment, but they die upon treatment with LCL161/TNF-α (10 μM; 10 nM) by cell death dependent on RIP1. This prediction was verified by Western blot detection of the apoptosis marker c-PARP (cleaved PARP, Figure 5A) and necroptosis marker pMLKL (phosphorylated MLKL protein, Figure 5B).

c-PARP was detected after 3 h treatment of WT cells with Eto (5 μM), Campt (1 μM), Taxol (0.1 μM), and LCL161/TNF-α (10 μM; 10 nM) and was absent in control untreated cells. In FADD-deficient cells also in control cells, only low level of c-PARP was present upon all treatments, indicating some basal level of c-PARP even in non-treated cells. In CASP3/7/6-deficient cells, no c-PARP signal was detected (Figure 5A).

Detection of c-PARP proved apoptosis induction especially in WT cells. To confirm our assumption that LCL161/TNF-α treatment induced necroptosis, we tried to detect necroptosis marker pMLKL (phosphorylated MLKL protein) after 24 h treatment with TNF-α, LCL161, and LCL161/TNF-α. The ratio of pMLKL to MLKL was evaluated. The most significant increase in pMLKL level was detected in CASP3/7/6-deficient cells upon combined treatment with LCL161/TNF-α. In FADD-deficient cells, response was more pronounced in TNF-α alone than in LCL161/TNF-α. No increase in pMLKL/MLKL ratio were detected in WT cells (Figure 5B). 

### 2.4. Surviving CASP3/7/6 and FADD-Deficient Cells Stop Dividing and Change Morphology

We performed time-lapse microscopy experiments on BioStation to study changes of cellular morphology of WT cells and CASP3/7/6- and FADD-deficient cells during long-time period of treatment (96 h) with selected anti-cancer drugs (Campt, Taxol, and Eto). Control WT cells (Figure S1) were round and divided regularly during the whole period. In the case of CASP3/7/6- (Figure S2) and FADD-deficient cells (Figure S3), we observed changes in morphology even in the control cells, which also divided, but in addition, they enlarged and changed the shape to elongated and/or amoeboid. This amoeboid morphology was highlighted upon the drug treatment. WT cells started dying during the first day of treatments with all drugs. At the beginning, dying cells displayed apoptotic morphology, while final state showed necrotic morphology (Figure S1). CASP3/7/6- and FADD-deficient cells (Figure S2 and Figure S3, respectively) remained alive during the whole time period, but stopped dividing, changed morphology, and enlarged. If the cells died, they showed necrotic morphology. These results show that CASP3/7/6- and FADD-deficient cells respond to drug treatment by arrest of cell proliferation, but most of the cells remain alive for at least 96 h. 

**Figure 6:**
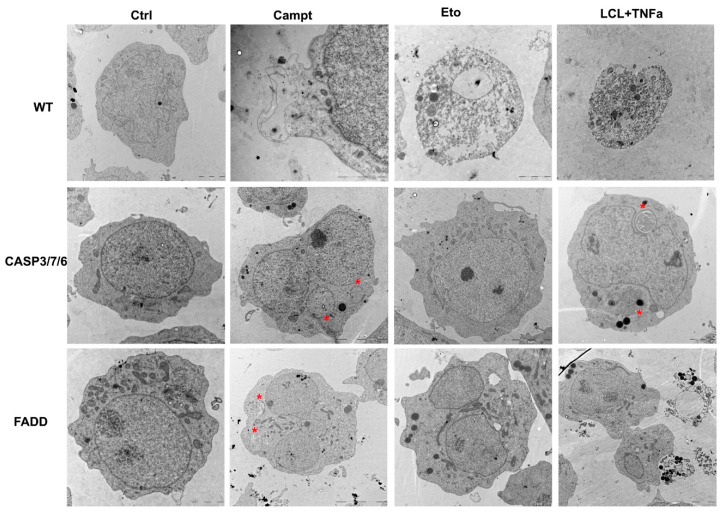
Electron microscopy of WT cells and CASP3/7/6- and FADD-deficient cells after 24 h treatment with Campt, Eto, and LCL161/ TNF-α (LCL)/TNF-a). In WT cells, there were only remains of dying and/or dead cells upon all treatments, showing necrotic morphology. Upon Campt (1 μM) treatment, cells were enlarged with lobate nuclei and contained autophagosomes (*) indicating induction of autophagy. Upon Eto (5 μM) and LCL161/TNF-α (10 μM; 10 nM) treatment, small number of mostly dead cells remained. On the contrary, CASP3/7/6- and FADD-deficient cells remained alive upon treatment with Campt and/or Eto and died only upon treatment with LCL161/TNF-α. Cells were enlarged and often contained multilobed nuclei. Mitochondria cristae disappeared upon Eto treatment. In the dead cells, we observed necrotic morphology. Bars 2 μm. In CASP3/7/6-deficient cells, Camp and Eto, and FADD-deficient cells, Campt and LCL161/TNF-α—bars = 5 μm.

For deeper insight into the cell ultrastructure, we performed transmission electron microscopy observation of WT cells and CASP3/7/6- and FADD-deficient cells after 24 h treatment with Campt, Eto, and LCL161/TNF-α. Dead cells upon all treatments revealed necrotic morphology. There were only residues of dying and/or dead cells upon all treatments in the case of WT cells due to the high cytotoxicity of all drugs. In contrast, CASP3/7/6- and FADD-deficient cells remained alive after treatment with Campt and/or Eto. These cells extensively died only upon treatment with LCL161/TNF-α. The tendency to engulf dead cells by neighboring cells was apparent. CASP3/7/6- and FADD-deficient cells were enlarged with lobate nuclei and contained autophagosomes indicating induction of autophagy upon Campt treatment. Mitochondria cristae were less prominent or disappeared after Eto treatment. All dead and dying cells showed typical necrotic morphology, i.e., damaged membranes, through which cellular content leaked (Figure 6). Electron microscopy is an end-point method and the results show the situation after 24 h treatment. It is possible that some cells, especially WT cells, underwent apoptosis in the first phase and apoptotic bodies were engulfed by surrounding cells.

## 3. Discussion

PCD is an important mechanism mediating cancer cell response to therapy. By studying the effect of common anti-cancer drugs on T-cell leukemia Jurkat cells with knocked out genes for key proteins of external apoptosis and necroptosis, we found out that FAS-associated death domain protein (FADD) and at least one of the effector caspases (i.e., 3, 6, and 7) are indispensable for the cell death execution. 

Our finding that the cells missing the activity of the effector caspases did not switch to non-caspase cell death indicates that caspase-dependent apoptosis is primarily activated upon treatment with anti-cancer drugs. Since also FADD is indispensable for cell death, apoptosis may be mediated via death receptors and/or via the cytoplasmic molecular complex ripoptosome. Ripoptosome was originally described as 2 MDa complex containing RIP1, FADD, and caspase 8, which assembles in the cytoplasm and regulates a switch between apoptosis and necroptosis by RIP1 kinase in response to the genotoxic stress and TLR3 stimulation [13,14] (Figure 1). 

Interestingly, regarding the members of the ripoptosome, only the absence of FADD significantly affected cell death in our experiments. On the contrary, the absence of caspase 8, which is normally part of the ripoptosome, did not reduce cellular mortality. This observation may be explained by the findings of other authors, showing that caspase 10 can alternate caspase 8 function in human cells. Caspase 10 is also a component of the ripoptosome, but its function in this complex has not been fully elucidated yet [13]. It is possible that caspase 10 plays an important role in the death of the cells with knocked out caspase 8. This our presumption is in accordance with observations of other authors. Lafont et al. provided the genetic evidence for the involvement of caspase 10 in FasL-induced cell death and additionally proved that z-VAD does not abrogate caspase 10 processing and cell death in Fas signaling [16]. The results of other authors also implicate the importance of caspase 10 in both extrinsic and intrinsic apoptosis pathways directed by ripoptosome [17,18,19]. It is likely that FADD is important for caspase 10 activation and that Jurkat cells in our experiments die by a mechanism involving caspase 10 and FADD. The involvement of caspase 10 in cell death initiation can also explain slight or no effect of pan-caspase inhibitor z-VAD in our experiments.

We demonstrated the resistance of FADD knocked out cells to treatment with anti-cancer drugs. On the contrary, double knocked out cells RIP3/FADD and MLKL/FADD were sensitive and RIP3/FADD revealed the highest sensitivity of all cell lines to treatment with anti-cancer drugs. These findings show that knockout of MLKL and especially knockout of RIP3- in FADD-deficient Jurkat cells can restore their ability to die and that RIP3 has a pro-survival role in the absence of FADD. In that case, the application of the RIP3 or MLKL inhibitors to the cells with non-functional FADD might support the effect of anti-cancer drugs on the principle of synthetic lethality. Abovementioned results could be explained by findings of authors showing that RIP3 negatively regulates apoptosis and its inhibition promotes cell death. Chang et al. described that RIP3 knockdown results in a shift from TNF-α-induced necroptosis to apoptosis in L929 cells [20]. In addition, RIP3 kinase inhibitors GSK’843 or GSK’872 activated caspases and triggered the apoptosis [21].

When both RIP3 and FADD are functional, FADD, as the key player of the ripoptosome, contributes to the activation of RIP1 and RIP3. Since the ripoptosome regulates both apoptosis and necroptosis and FADD is additionally a member of several other cell death complexes, it is possible that FADD is involved in both apoptosis and necroptosis. This role of FADD was described by many authors and is summarized at [22,23]. However, studies dealing with the role of FADD in necroptosis show controversial results. Several groups have previously reported that FADD is involved in triggering non-apoptotic cell death [24,25]. Irrinki et al. observed resistance of MEF cells with knocked out FADD to TNF-α-induced necroptosis [26]. Holler et al. (2000) showed that Fas kills activated primary T-cells in the absence of active caspases by cell death dependent on FADD or receptor-interacting protein (RIP) [27]. RIP1 is also required for necrotic death induced by tumor necrosis factor (TNF) and TRAIL. FADD can bind to RIP1 and RIP3 and might directly modulate the interaction between them, therefore it could be involved in regulation of necroptosis [10,23]. On the other hand, some authors demonstrated negative role of FADD in TNF-induced necroptosis. A study on L929 cells showed that FADD depletion induced necroptosis [28]. In this work, we demonstrate that cell death of Jurkat cells upon treatment with several anti-cancer drugs is FADD dependent. However, treatment with TNF-α/SM enabled FADD-deficient Jurkat cells to die by RIP1-dependent necroptosis. This our result is identical to the result of Feldmann et al., who also studied FADD-deficient Jurkat cells [29]. The different effect of anti-cancer drugs and TNF-α/SM treatment on FADD-deficient cells shows that the response of cells depends not only on the activity of signaling pathways but also on the drug used. We propose that cytoplasmic complex ripoptosome regulates cell death via two alternative pathways, one involving caspase 8 (alternated by caspase 10) and the other via RIP kinases but both dependent on FADD (Figure 1).

Based on our results, we were wondering whether it is possible to induce cell death in cells that were resistant to all our initial treatments, i.e., CASP3/7-, CASP3/7/6-, and FADD-deficient cells. Therefore, we tried to trigger external apoptosis or necroptosis through the TNFR1 death receptor using cytokine TNF-α. TNF-α significantly increased toxicity of Eto in CASP3/7- and FADD-deficient cells, while the effect on CASP3/7/6-deficient cells was only moderate. Different responses of CASP3/7- and CASP3/7/6-deficient cells indicate that the effector caspase 6 is able to substitute the missing caspases 3 and 7. Significance of the caspase 6 in the absence of caspases 3 and 7 was described also by Wang et al. [9].

Based on the findings that combined treatment with SM and TNF-α induces necroptosis in cells with inhibited caspases [11,30], we tried to kill immortal FADD- and CASP3/7/6-deficient cells by TNF-α/LCL161. In our experiments, TNF-α in combination with LCL161 increased the mortality of both CASP3/7/6- and FADD-deficient cells, but also the mortality of other knocked out cells with the exception of RIP1-deficient cells. These results demonstrate that cell death of CASP3/7/6- and FADD-deficient cells upon TNF-α and LCL161 treatment is RIP1 dependent. We proved this presumption also using RIP1 inhibitor Nec-1, which significantly reduced dying of these cells in contrast to slightly effective caspase inhibitor z-VAD. In addition, we demonstrated necroptotic character of this cell death by detection of phosphorylated MLKL protein, main effector of necroptosis, and by morphological changes observed in electron microscopy. We propose that LCL161 induces ripoptosome formation due to deubiquitination of RIP1. Our results are consistent with results McComb et al., who described the importance of RIP1 in response to SM (birinapant) on Jurkat and other leukemia cells [31]. These authors demonstrated that cells could switch to an alternative death pathway when a single pathway is inhibited. Nec-1 (RIP1 inhibitor) affected cell death upon most treatments. In addition, Abhari et al. identified RIP1 as a critical mediator of the synergistic interaction of IAPs inhibitors together with TRAIL receptor agonists on neuroblastoma cells [32]. Key role of RIP1 in response to combined treatment with SM and several anti-cancer drugs was observed by Loder et al. in various ALL cells [33]. Wang et al. demonstrated the effect of Nec-1 on TNF-α and SM treatments [34]. Laukens et al. in accordance with our results observed that SM and TNF-α kill FADD and also caspase 8-deficient Jurkat cells by necroptosis, but this treatment induced apoptosis in WT cells [35]. In agreement with our prediction, all these authors proved the importance of RIP1 and the formation of a RIP1/FADD/caspase 8 complex as a critical regulator of this synergism.

When interpreting and generalizing the results, the properties of the cells on which the experiments were performed should be considered. Jurkat cells, like other tumor cells, have specific properties and their response to the drugs may be unique. In general, there are two apoptotic signaling pathways activated by ligation of the death receptors. In type I cells signaling involves ligand-induced recruitment of FADD and procaspase 8 to DISC complex, leading to direct activation of caspase 3. In type II cells, DISC complex is formed to less extent and caspase 8 cleaves the pro-apoptotic protein Bid to yield a truncated Bid (tBid). tBid acts in dependence on Bcl-2 or Bcl-xL on the mitochondria to promote the release of cytochrome c [36]. Despite Jurkat cells do not express Bax and p53, they belong to type II group of cells, which activates mitochondrial pathway. In these cells, activation of the Fas receptor produces the release of cytochrome c because of induction of the mitochondrial permeability transition (MPT) [37]. However, it was found out that in Jurkat cells upon Eto treatment full-length Bid, but not tBid, is translocated from the cytosol to the mitochondria with the consequent induction of the MPT and that Jurkat cells utilize p73 and Bid instead of p53 and Bax for cytochrome c release [37]. Considering these findings, it is clear that executioner caspases are required for triggering of apoptosis in our experiments. However, it is very surprising that the FADD protein, usually involved in the external pathway signaling, is also indispensable. This is consistent with our assumption that ripoptosome plays an important role in triggering cell death by anti-cancer drugs.

It is apparent that PCD signaling pathways are linked by key players, whose functions are not strictly limited to one cell death modality. Thus, significant effectivity of Nec-1 could be due to its ability to inhibit not only necroptosis but also apoptosis [7,38]. 

Collectively, our results show that common anti-cancer drugs trigger primarily apoptosis dependent on executioner caspases and FADD. Interestingly, CASP3/7/6- and FADD-deficient cells are unable to switch to necroptosis and are resistant. Nonetheless, combined treatment with TNF-α and smac mimetic partially overcomes drug resistance via RIP1-dependent necroptosis. Thus, a combination of TNF-α and smac mimetic could be a suitable strategy for overcoming resistance to therapy in cells unable to trigger apoptosis.

## 4. Materials and Methods

### 4.1. Reagents

Camptothecin, vinblastine, Taxol, TNF-α, propidium iodide, and ECL were purchased from Sigma-Aldrich, Darmstadt, Germany; etoposide was purchased from Alexis Biochemicals, Farmingdale, NY, USA; doxorubicin was purchased from Alchimica, Prague, Czech Republic; and necrostatin-1 (Nec-1), z-VAD-fmk (z-VAD), LCL161, and dinaciclib were purchased from Selleckchem, Houston, TX, USA.

### 4.2. Cell Lines and Cultivation Conditions

Jurkat WT cells and various single- and double-knockout lines derived from them (RIP1, RIP3, CASP8, CASP3, CASP6, CASP3/7, CASP3/7/6, MLKL, MLKL/CASP8, FADD, MLKL/FADD, and RIP3/FADD) were obtained from the laboratory of Dr. Bornhauser at the University Children’s Hospital Zurich. The lines were established using multicolor lentiCRISPR plasmids as described previously [31]. The cells were grown in RPMI 1640 medium supplemented with 2 mM glutamine and 10% fetal calf serum, 100 IU/mL penicillin, and 100 µg/mL streptomycin (PAA Laboratories, Pasching, Austria). Cells were incubated at 37 °C under 5% CO_2_ in a high-humidity atmosphere and subcultured three times a week.

### 4.3. Propidium Iodide Exclusion Assay

The cell viability assay was based on the exclusion of propidium iodide (PI) by the intact viable cells. The cells were plated in 24-well tissue culture test plates (Orange Scientific, Braine-I’Alleud, Belgium) at 5 × 10^4^ cells/mL and treated with tested compounds alone or in combination with the inhibitors. These compounds and inhibitors were used: camptothecin (Campt, 1 μM), etoposide (Eto, 5 μM), doxorubicin (Doxo, 0.15 μM), vinblastine (vinbl, 0.1 μM), Taxol (0.1 μM), dinaciclib (Dina, 40 nM), TNF-α (10 ng/mL), LCL161 (10 μM), z-VAD-fmk (z-VAD, 20 μM), and necrostatine-1 (Nec-1, 20 μM). Anti-cancer drug concentrations were chosen based on testing different concentrations of drugs on WT cells. The concentration that killed about 60–80% of the cells was selected for further experiments. PI was added after 48 h of incubation and immediately after PI addition, the percentage of dead (PI-positive) cells was detected using Cytomics FC 500 flow cytometry system (Beckman Coulter, Inc., CA, USA) using channel FL3 (emission at 620 nm). A total of 10,000 cells was analyzed for each sample. Each experiment included two replicate wells and was repeated at least three times.

### 4.4. SDS-PAGE and Western Blot Analysis

Cells were seeded at the concentration of 10^5^ cells/mL and treated with tested compounds or their combination with the inhibitors for 3 or 24 h. After the treatment, cells were lysed in 2x Laemmli buffer. Whole-cell lysates were separated by electrophoresis on 10% SDS-PAGE gels and proteins were transferred onto methanol pre-treated PVDF membranes (Millipore, Billerica, MA, USA). Membranes were blocked with 5% non-fat dry milk (for MLKL, PCNA, and c-PARP antibody) and 2% bovine serum albumin (BSA, Sigma for pMLKL antibody) in TBS (10 mM TRIS-HCl, 100 nM NaCl, and 0.05% Tween 20; pH = 7.4) for 1 h at room temperature and incubated with primary antibodies (dilutions recommended by the manufacturer) at 4 °C overnight, followed by 1 h incubation with mouse antirabbit IgG HRP and goat antirat IgG HRP (sc- 2357, sc2006, Santa Cruz Biotechnology). The following primary antibodies were used: mouse anti-MLKL (MABC604), mouse anti-phospho MLKL (Thr357, ABC234, Merc), and rabbit anti-c-PARP mAb (Cell Signaling #5625). Anti-PCNA (PC-10) mouse mAb was kindly provided by Bořivoj Vojtěšek (Masaryk Memorial Cancer Institute, Brno, Czech Republic). Signal was developed with an ECL chemiluminescence reagent and detected using G:BOX (Syngene, Bengaluru, India).

### 4.5. Long Time Live Cell Imaging 

BioStation CT (Nikon Instruments Inc., Melville, NY, USA) was used for long-time observation of drugs treated cells using phase-contrast microscopy. Cells were seeded to Poly-L-Lysin coated 24-well plates in 300 μL at density 10^5^/mL and cultivated upon conditions described above. Phase-contrast objective 10× was used. The movies were recorded in phase-contrast mode at 15 min intervals for 96 h. Microscopy images were processed by ImageJ and Adobe Photoshop software. 

### 4.6. Transmission Electron Microscopy

WT cells and CASP3/7/6-, and FADD-deficient cells were treated with Campt (1 μM), Eto (5 μM), and LCL/TNF-α (10 μM; 10 nM) for 24 h, collected, washed three times in 0.1 M cacodylate buffer (pH = 6.98), fixed in a 3% glutaraldehyde in 0.1 M cacodylate buffer (containing 0.2 M saccharose) for 60 min, and post-fixed in 1% osmium tetroxide in 0.1 M cacodylate buffer for 45 min at room temperature. After rapid dehydration in a graded series of ethanol, the cells were embedded in Durcupan. Ultrathin sections prepared with a diamond knife on an Ultracut Reichert-Jung ultramicrotome were placed on formvar-coated copper grids, stained with 2.5% uranyl acetate (6 min) and lead citrate (3 min), and observed using a transmission electron microscope Philips Morgagni (FEI Company, Brno, Czech Republic).

### 4.7. Statistical Analysis

At least three independent experiments were performed under identical conditions. Data are expressed as the means ± SD. Results were analyzed using the Student´s *t*-test, and significance differences are *p* < 0.05; *p* < 0.01; *p* < 0.005.

## 5. Conclusions

There are several important implications of this study. First, conventional anti-cancer drugs induce primarily apoptosis in T-cell leukemia Jurkat cells and the knockout of the FADD or the combined knockout of all executioner caspase genes (i.e., caspase 3, 6, and 7) disables cell death upon common anti-cancer drugs treatments. These cells remain alive, but non-dividing. Second, FADD- and/or CASP3/7/6-deficient cells die upon combined treatment with SM (LCL161) and TNF-α. Third, the cell death triggered by TNF-α/SM is RIP1-dependent necroptosis. Fourth, despite RIP1 is essential for cellular response to TNF-α/SM treatment, it is completely dispensable in the response to anti-cancer drugs. Fifth, the individual caspases, either initiator (caspase 8) or effector (caspase 3 or 6) caspases, are dispensable for the cell death execution, indicating the interchangeability of the particular caspases. Based on these results, we speculate that ripoptosome, a cytoplasmic complex consisting of RIP1, FADD, and caspase 8 [10], plays a crucial role in the cell death signaling upon treatment with the anti-cancer drugs.

Taken together, we show the key role of FADD and caspases in a leukemia cells response to treatment with various anti-cancer drugs. Combined treatment with TNF-α/SM allows resistant cells to switch from apoptosis to RIP1-dependent necroptosis. These findings could have implications for treatment of drug-resistant cancers.

## Data Availability

All data are in manuscript and Supplementary Materials.

## Supplementary Materials

The following are available online at https://www.mdpi.com/1422-0067/22/5/2702/s1, Figure S1: Serial images showing WT cells control (A) and upon treatment with Campt (1 μM, B), Taxol (0.1 μM, C), and vinbl (0.1 μM, D) obtained from BioStation time-lapse microscopy. The videos were recorded using 10× objective in phase-contrast mode at 15 min intervals for 96 h. * apoptotic morphology. Highlighted is apoptotic cell. Figure S2: Serial images showing CASP3/7/6-deficient cells control (A) and upon treatment with Campt (1 μM, B), Taxol (0.1 μM, C), and vinbl (0.1 μM, D) obtained from BioStation time-lapse microscopy. The videos were recorded using 10× objective in phase-contrast mode at 15 min intervals for 96 h. Highlighted are cells with amoeboid morphology. Figure S3: Serial images showing FADD-deficient cells control (A) and upon treatment with Campt (1 μM, B), Taxol (0.1 μM, C), and vinbl (0.1 μM, D) obtained from BioStation time-lapse microscopy. The videos were recorded using 10× objective in phase-contrast mode at 15 min intervals for 96 h. Highlighted are cells with amoeboid morphology.
