# Supplementary figures and images for: Loss of FADD and Caspases Affects the Response of T-Cell Leukemia Jurkat Cells to Anti-Cancer Drugs
Source: Int J Mol Sci. 2021 Mar 7;22(5):2702. doi: 10.3390/ijms22052702 (PMC7962194; doi:10.3390/ijms22052702)

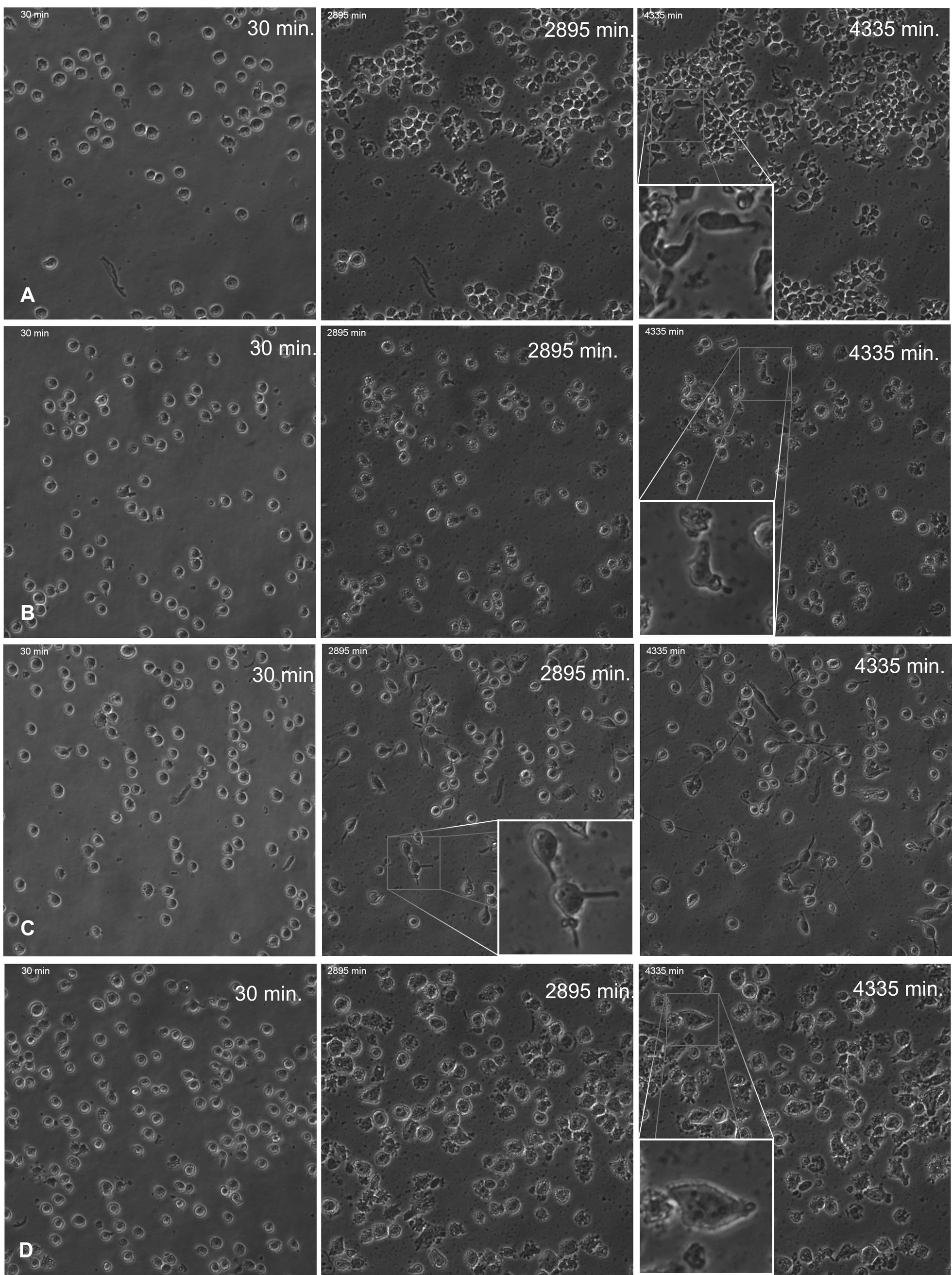

Supplement: Supplementary file 1 [file ijms-22-02702-s001.zip › FigS3.tif]

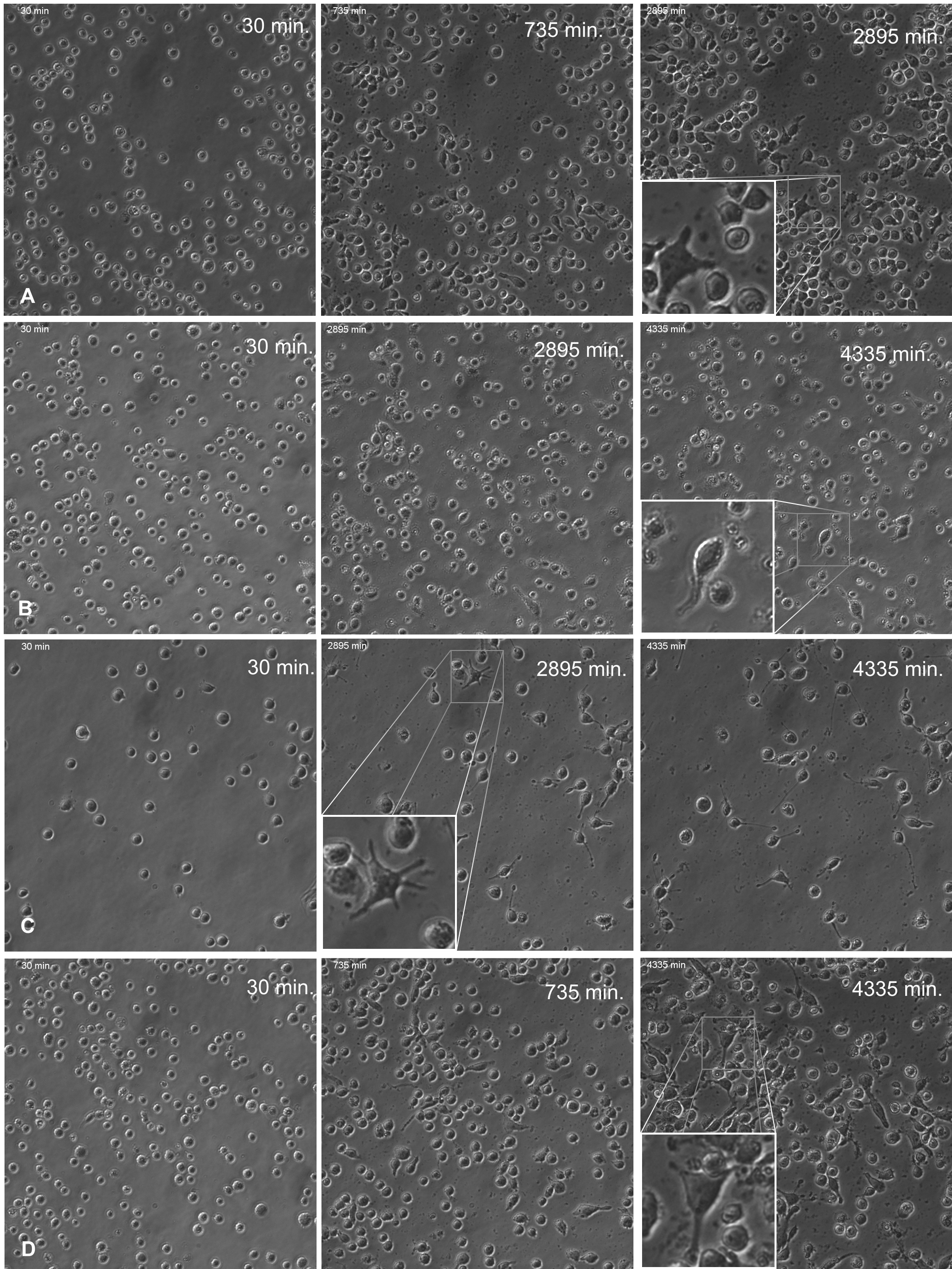

Supplement: Supplementary file 1 [file ijms-22-02702-s001.zip › FigS2.tif]

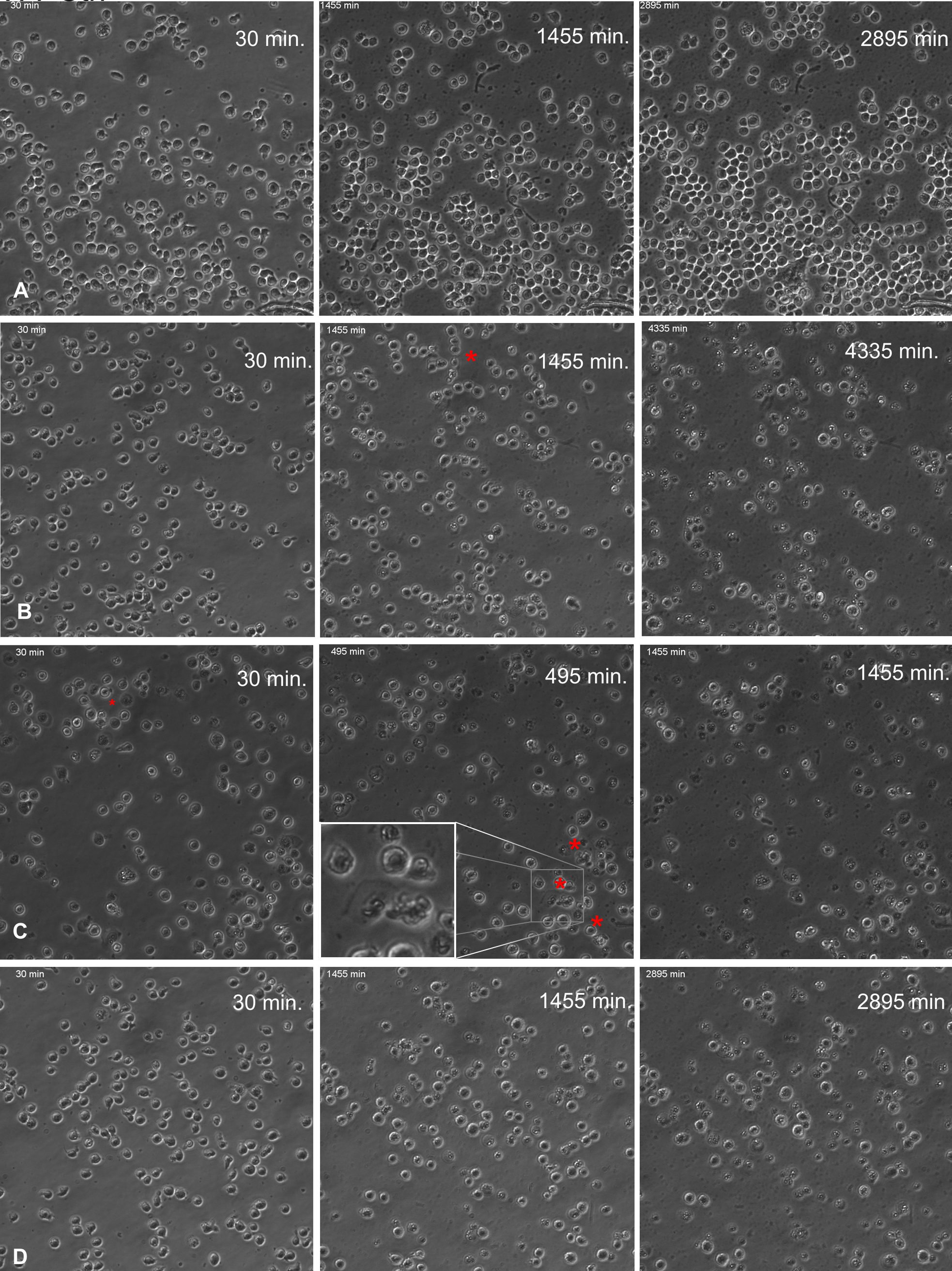

Supplement: Supplementary file 1 [file ijms-22-02702-s001.zip › FigS1.tif]
